# Synthesis and comparative evaluation of ^177^Lu-labeled PEG and non-PEG variant peptides as HER2-targeting probes

**DOI:** 10.1038/s41598-022-19201-9

**Published:** 2022-09-20

**Authors:** Amit Kumar Sharma, Rohit Sharma, Kusum Vats, Haladhar Dev Sarma, Archana Mukherjee, Tapas Das, Drishty Satpati

**Affiliations:** 1 Radiopharmaceuticals Division, Bhabha Atomic Research Centre, Mumbai, India; 2 Homi Bhabha National Institute, Mumbai, India; 3 Radiation Biology and Health Sciences Division, Bhabha Atomic Research Centre, Mumbai, India

**Keywords:** Peptides, Molecular medicine, Breast cancer

## Abstract

Highest global cancer incidence of female breast cancer is a matter of great concern. HER2-positive breast cancers have high mortality rate hence detection at an early stage is vital for successful treatment, improved cancer care and survival rate. Radiolabeled peptides have emerged as new alternatives to radiolabeled antibodies to overcome the limitations of slow clearance and uptake in non-target tissues. Herein, DOTA-A9 peptide and its pegylated variant were constructed on solid phase and radiolabeled with [^177^Lu]LuCl_3_. [^177^Lu]DOTA-A9 and [^177^Lu]DOTA-PEG_4_-A9 displayed high binding affinity (K_d_ = 48.4 ± 1.4 and 55.7 ± 12.3 nM respectively) in human breast carcinoma SKBR3 cells. Two radiopeptides exhibited renal excretion and rapid clearance from normal organs. Uptake in SKBR3 tumor and tumor-to-background ratios were significantly higher (p < 0.05) for [^177^Lu]DOTA-PEG_4_-A9 at the three time points investigated. Xenografts could be clearly visualized by [^177^Lu]DOTA-PEG_4_-A9 in SPECT images at 3, 24 and 48 h p.i. indicating the potential for further exploration as HER2-targeting probe. The encouraging in vivo profile of PEG construct, [^177^Lu]DOTA-PEG_4_-A9 incentivizes future studies for clinical applications.

## Introduction

With the ascent in the global cancer burden considerable rise in the female breast cancer worldwide has been recorded by the Global Cancer Statistics 2020. The global cancer incidence of female breast cancer (11.7%) has even surpassed the earlier most commonly diagnosed lung cancer (11.4%)^1^. Magnitude of the disease however can be controlled by focusing on rapid detection and the affiliated treatment to the affected individuals. Identification and development of targeted smart probes is the key to stimulate diagnosis and therapy leading to better management of breast cancer patients. Most commonly diagnosed breast cancers are hormone-receptor positive whereas human epidermal growth factor receptor 2 (HER2)-positive cancers are less frequent (15–20%). However, HER2-positive breast cancers are more aggressive and critically associated with metastatic spread and poor prognosis^2–5^. HER2-targeted humanized monoclonal antibodies trastuzumab and pertuzumab have been approved by the Food and Drug Administration (FDA) for breast cancer treatment^6,7^. High molecular weight (~ 150 kDa) and long biological half-life of antibodies increases residence time in the blood posing severe limitation during nuclear medicine imaging studies (extended imaging time periods, poor quality and high radiation burden)^8–10^. Hence the recent trend has been development of low molecular weight constructs like peptides for clinical applications due to their faster excretion, rapid target penetration and better image contrast in short duration. Peptides exhibiting appropriate affinity and specificity towards the target, flexibility, facile synthetic protocol and favorable pharmacokinetics can be radiolabeled with different radionuclides for diversified use as imaging/ therapeutic/ theranostic probes. Radiolabeled peptides can thus be recommended to be propitious alternative for HER2-targeted imaging and therapy^11–13^. The few reported radiolabeled HER2-binding peptides are; KCCYSL (In-111, Cu-64, Ga-68), LTVSPWY (Tc-99m, Ga-68), YLFFVFER (H6F) and KLRLEWNR (H10F) (Tc-99m), FCGDFYACYMDV (AHNP) (In-111) and QDVNTAVAW (A9) (In-111)^14–22^.

Though several peptide-based HER2-targeting probes are being explored and studied under pre-clinical settings and investigated, low binding affinity, low tumor levels and tumor to organ ratios has restricted the clinical applications of radiopeptides. Hence more focused research and efforts are needed to develop promising HER2-targeted peptides for clinical nuclear imaging purpose. In this study we investigated the peptide A9 which has been derived from the Trastuzumab-Fab portion and reported to display high HER2 affinity. Hence the A9 peptide was synthesized, modified at the N-terminus and conjugated with 1,4,7,10-Tetraazacyclododecane-1,4,7,10-tetraacetic acid (DOTA) chelator for incorporating [^177^Lu]LuCl_3_ radionuclide. Lu-177 labeled peptides serve the dual purpose of imaging and therapy and therefore can be used as theranostic probes. Particulate emissions (E_β_^-^_(av)_ = 134 keV, E_βmax_ = 497 keV) along with half-life of 6.6 days and average range of 0.6 mm in soft tissues are the ideal characteristics of [^177^Lu]LuCl_3_ for therapeutic applications. Simultaneous emission of γ photons with energies 113 keV (6.4%) and 208 keV (11%) is the added advantage for imaging purpose and online monitoring of the patient^23^.

Small peptides often suffer from breakdown in the blood by peptidases/proteases leading to quick elimination from the body and thereby curtailing the circulation time and the probability of binding to the target. Designing of peptides with hydrophilic and flexible polyethylene glycol (PEG) chains leads to extended circulation and proteolytic resistance thereby improving tumor targeting capability, pharmacokinetic profile and clinical efficacy of peptides^24^. Hence the present study aimed at constructing two variants of peptide A9. First version was simply coupled with DOTA at N-terminus whereas a polyethylene glycol (PEG) moiety was introduced at the N-terminus of the second variant before DOTA conjugation. Secondary structure of peptides was examined by CD spectroscopy in the far-UV region. The two A9 variants were radiolabeled with [^177^Lu]LuCl_3_ and tested in vitro in HER2-expressing human breast carcinoma SKBR3 cancer cells. Tumor targeting efficiency and pharmacokinetics of the two radiopeptides was investigated by performing biodistribution and SPECT imaging studies in subcutaneous SKBR3 tumor bearing mouse xenografts.

## Results

### Chemistry and radiochemistry

**Figure 1:**
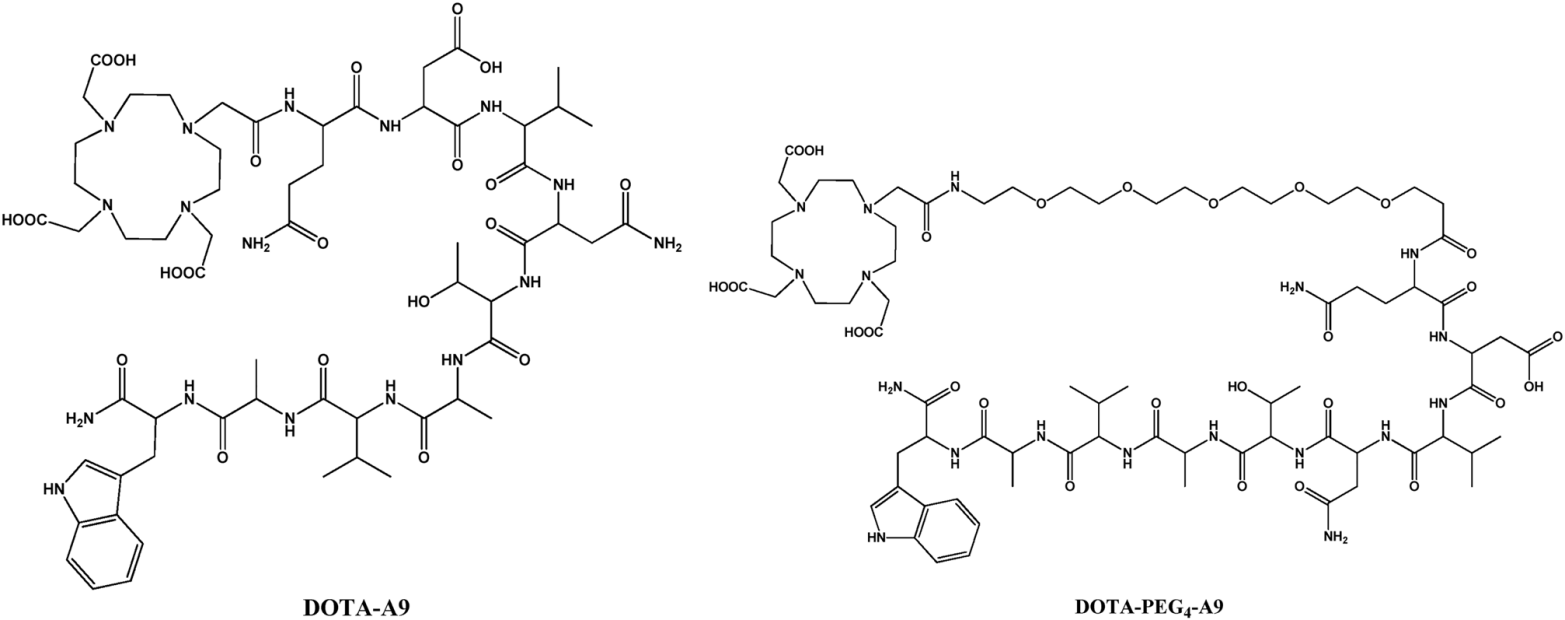
Chemical structures of peptides DOTA-A9 and DOTA-PEG_4_-A9.

Peptides: Gln-Asp-Val-Asn-Thr-Ala-Val-Ala-Trp (A9) and PEG_4_-Gln-Asp-Val-Asn-Thr-Ala-Val-Ala-Trp (PEG_4_-A9) were synthesized on NovaSyn TGR resin using classical Fmoc solid phase peptide synthesis (SPPS) chemistry protocol. Chelator DOTA was introduced at the N-terminus of peptides following the methodology similar to that used for coupling of amino acids (Fig. 1). Subsequent to cleavage from the resin peptides were purified and lyophilized. The yield of peptides was ~ 15% and purity as determined from 220 nm UV-trace was > 98%. Identity of DOTA-peptides was confirmed by MALDI-TOF spectrometry as shown in the (Supplementary Fig. S1). HPLC retention times of DOTA-A9 and DOTA-PEG_4_-A9 peptides were 15.1 and 16.3 min respectively.

**Figure 2:**
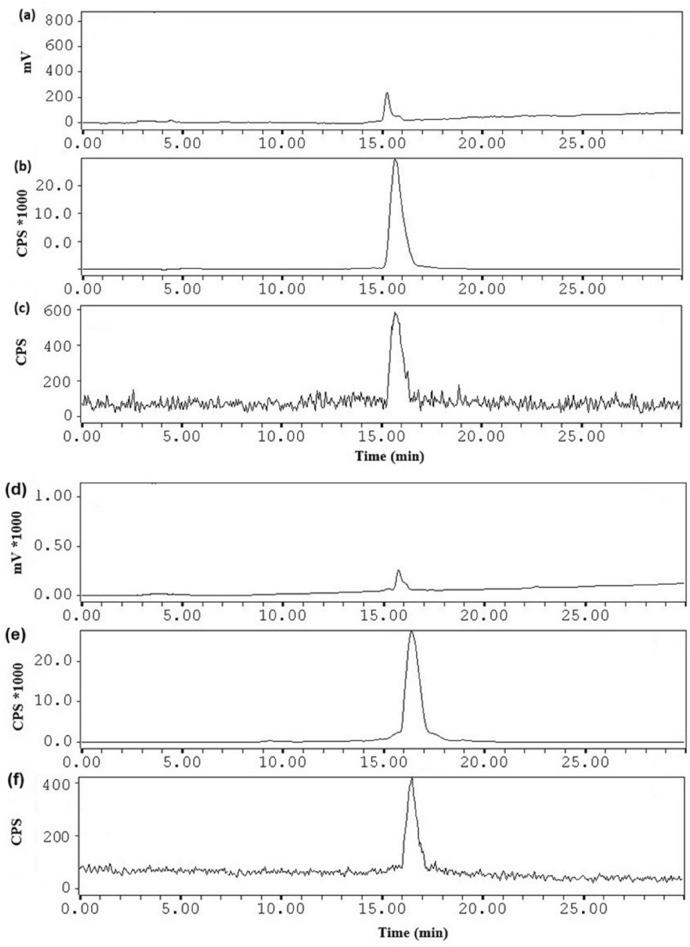
Analytical HPLC profile (**a**) UV-chromatogram (λ = 220 nm), (**b**) radiochromatogram of freshly prepared ^177^Lu-DOTA-A9, (**c**) radiochromatogram of human serum after 6 h incubation with ^177^Lu-DOTA-A9 and analytical HPLC profile, (**d**) UV-chromatogram (λ = 220 nm), (**e**) radiochromatogram of freshly prepared ^177^Lu-DOTA-PEG_4_-A9, (**f**) radiochromatogram of human serum after 6 h incubation with ^177^Lu-DOTA-PEG_4_-A9.

Purified and characterized DOTA-A9 and DOTA-PEG_4_-A9 peptides were radiolabeled with [^177^Lu]LuCl_3_ in > 98% radiochemical yield and purity as determined by paper chromatography (ACN/H_2_O, 1:1, v/v) and radio-HPLC chromatograms respectively. RP-HPLC retention time of [^177^Lu]DOTA-A9 was 15.8 min and no degradation on incubation with human serum was observed as shown in the (Fig. 2). [^177^Lu]DOTA-PEG_4_-A9 exhibited retention time of 16.5 min with no change in radiochromatogram after 6 h incubation with human serum as shown in the (Fig. 2). Specific activity of Lu-177 labeled peptides was 30 ± 5 GBq/µmol. Radiopeptides were highly hydrophilic according to the partition coefficient (log P_o/w_) values of radiopeptides {[^177^Lu]DOTA-A9: − 3.29 ± 0.1; [^177^Lu]DOTA-PEG_4_-A9: − 3.45 ± 0.1}. Since radiopeptides were prepared in high quantitative yield they were used without purification for in vitro and in vivo studies.

### Circular dichroism

The CD spectrum of the two peptides is displayed in (Supplementary Fig. S2). The two peptides, DOTA-A9 and DOTA-PEG_4_-A9 exhibited nearly similarly spectra with negative peak at 215 nm and 218 nm respectively. Positive peak for [^177^Lu]DOTA-PEG_4_-A9 was observed at 198 nm. The peaks are characteristic of β-sheet structure^27^.

### In vitro cell studies

Specific cellular uptake exhibited by [^177^Lu]DOTA-A9 and [^177^Lu]DOTA-PEG_4_-A9 in HER2 over-expressing SKBR3 cells was 6.1 ± 0.4% and 3.08 ± 0.1% respectively. Uptake of [^177^Lu]DOTA-A9 and [^177^Lu]DOTA-PEG_4_-A9 in HER2-negative, MDA-MB-231 cells was 0.28 ± 0.05% and 0.39 ± 0.15% respectively. Binding affinity of the two radiopeptides was in nanomolar range and nearly similar for [^177^Lu]DOTA-A9 and [^177^Lu]DOTA-PEG_4_-A9 (K_d_ = 48.37 ± 1.37 nM and 55.67 ± 12.3 nM respectively). Our group previously reported the binding affinity of [^177^Lu]DOTA-Trastuzumab in SKOV3 cells (K_d_ 13.61 nM, B_max_ 31.09 pmol per 10^6^ cells)^28^. The B_max_ value for [^177^Lu]DOTA-A9 and [^177^Lu]DOTA-PEG_4_-A9, expressed as the number of HER2 receptors per cell, was calculated to be 1.8 × 10^6^ and 6.6 × 10^5^ respectively.

In SKBR3 spheroids, maximum uptake of [^177^Lu]DOTA-A9 and [^177^Lu]DOTA-PEG_4_-A9 was 1.67 ± 0.16 and 0.56 ± 0.06% respectively while in MDA-MB-231 spheroids the corresponding values were 0.78 ± 0.3 and 0.70 ± 0.2%. No statistically significant difference between the uptake of [^177^Lu]DOTA-PEG_4_-A9 in HER2-positive and HER2-negative spheroids was observed. However, there was a statistically significant difference (p < 0.05) between the uptake of [^177^Lu]DOTA-A9 in HER2-positive and HER2-negative spheroids.

### Biodistribution studies

**Figure 3:**
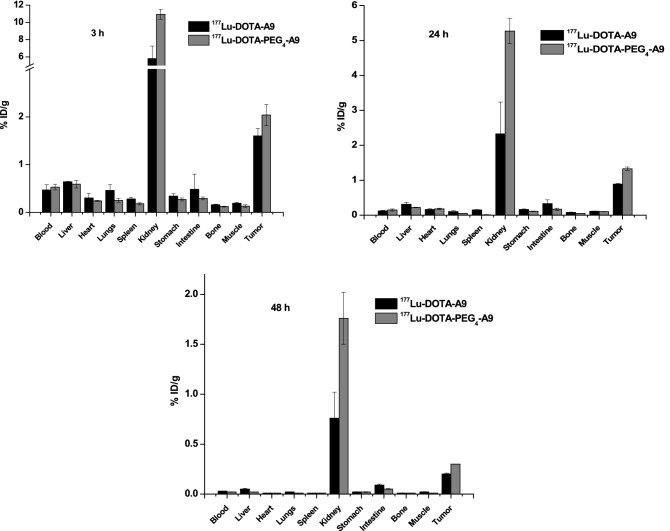
Comparative biodistribution results of ^177^Lu-DOTA-A9 and ^177^Lu-DOTA-PEG_4_-A9 at 3 h, 24 h and 48 h p.i. in female SCID mice bearing SKBR3 tumors. Error bars represent standard deviation.

Biodistribution results of [^177^Lu]DOTA-A9 and [^177^Lu]DOTA-PEG_4_-A9 at different time points (3, 24 and 48 h p.i.) in SKBR3 tumor bearing mouse xenografts are presented in (Fig. 3). Tumor uptake values of [^177^Lu]DOTA-PEG_4_-A9 were significantly higher than of [^177^Lu]DOTA-A9 at the three time points investigated (p < 0.05 for 3 h and p < 0.005 for 24 h and 48 h). The two radiopeptides demonstrated significantly higher (p < 0.05) accumulation of radioactivity in the tumor in comparison to other normal organs (heart, lungs, liver, intestine, stomach, spleen and muscle).

**Figure 4:**
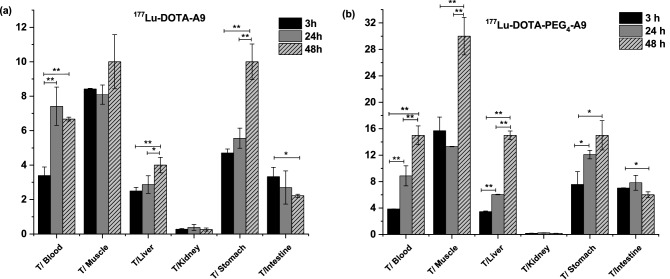
Tumor to background ratios of (**a**) ^177^Lu-DOTA-A9 and (**b**) ^177^Lu-DOTA-PEG_4_-A9 at 3 h, 24 h and 48 h p.i. Error bars represent standard deviation. *p < 0.05 and **p < 0.005.

Tumor-to-background ratios for [^177^Lu]DOTA-A9 and [^177^Lu]DOTA-PEG_4_-A9 at 3, 24 and 48 h p.i. are presented in (Fig. 4a,b) respectively. Tumor-to-blood ratio of [^177^Lu]DOTA-A9 increased from 3.40 ± 0.49 at 3 h to 7.42 ± 1.11 at 24 h but there was no further improvement at 48 h. Ratio however significantly increased (p < 0.005) for [^177^Lu]DOTA-PEG_4_-A9 from 3 h (3.85 ± 0.02) to 24 h (8.87 ± 1.51) and 48 h (15 ± 1.42). High tumor-to-muscle ratio was observed for [^177^Lu]DOTA-A9 at 3 h (8.42 ± 0.04) which remained similar at 24 h with slight improvement at 48 h (10 ± 1.57).

**Figure 5:**
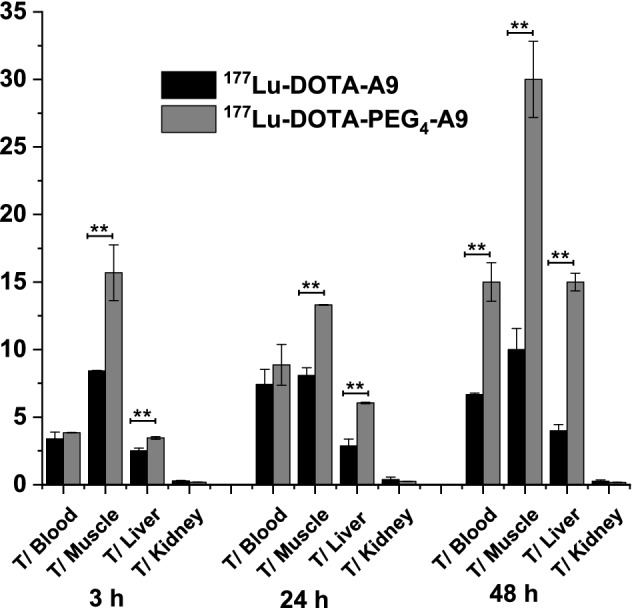
Comparative tumor-to-blood, tumor-to-muscle, tomor-to-liver and tumor to kidney ratios of ^177^Lu-DOTA-A9 and ^177^Lu-DOTA-PEG_4_-A9 at 3 h, 24 h and 48 h p.i. Error bars represent standard deviation. *p < 0.05 and **p < 0.005.

Comparative tumor-to-blood, tumor-to-muscle, tumor-to-liver and tumor-to-kidney ratio for the two radiopeptides at 3, 24 and 48 h p.i. are presented in (Fig. 5). [^177^Lu]DOTA-PEG_4_-A9 displayed significantly higher (p < 0.005) tumor-to-muscle ratio than [^177^Lu]DOTA-A9 at 3 h (15.69 ± 2.06) which further increased to 30 ± 2.83 at 48 h. Tumor-to-muscle, tumor-to-liver, tumor-to-stomach and tumor-to-intestine values were also significantly higher (p < 0.05) for [^177^Lu]DOTA-PEG_4_-A9 in comparison to [^177^Lu]DOTA-A9 at all the time points examined. Predominant excretion route of the two radiopeptides was through urine-renal pathway. Significantly higher kidney uptake (p < 0.05) was observed for [^177^Lu]DOTA-PEG_4_-A9 compared to [^177^Lu]DOTA-A9 at the three time points leading to lower tumor-to-kidney ratio for the pegylated variant.

### SPECT imaging

**Figure 6:**
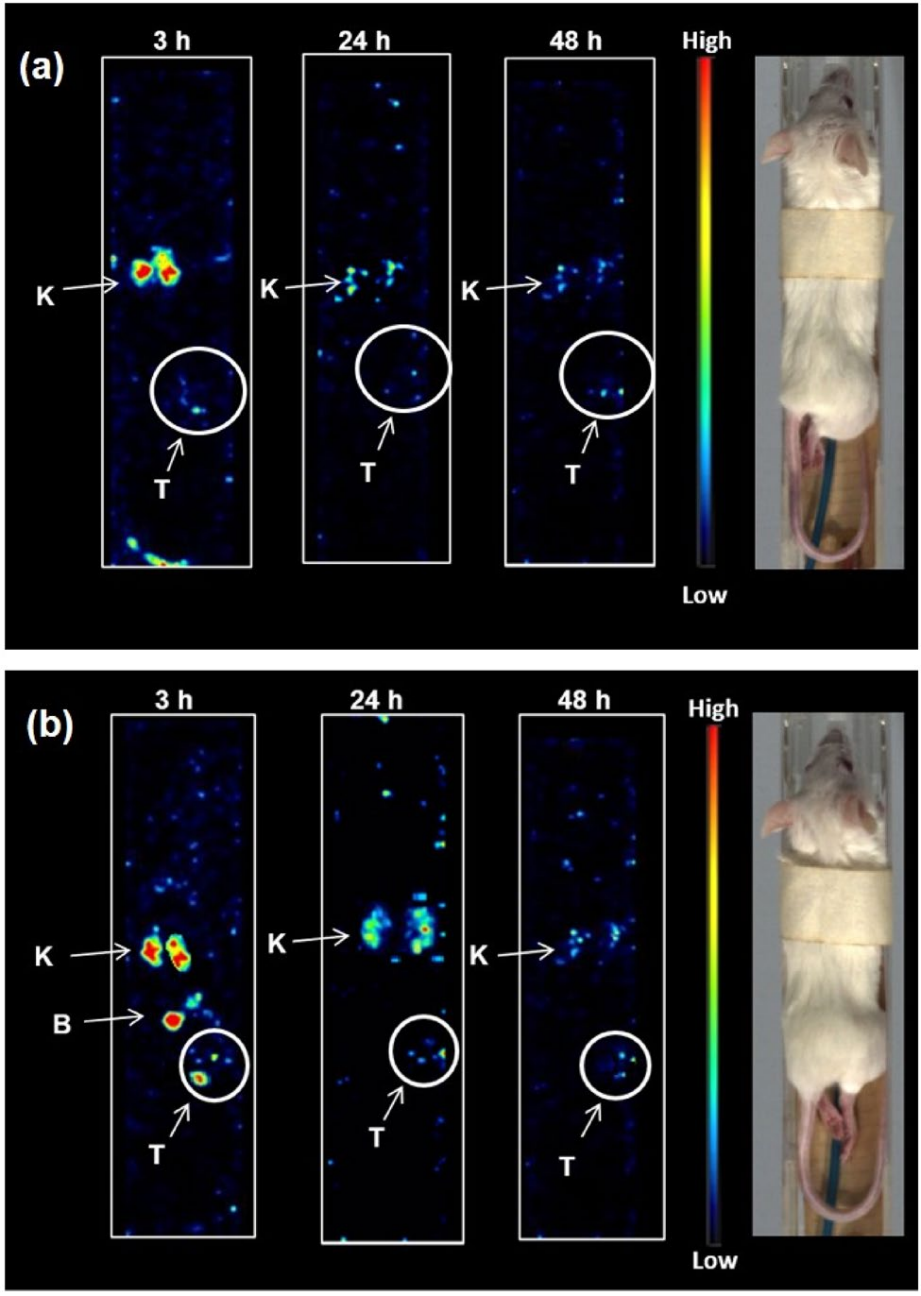
SPECT images of female SCID mice bearing SKBR3 tumor xenografts at 3 h, 24 h and 48 h p.i. injected with (**a**) ^177^Lu-DOTA-A9 and (**b**) ^177^Lu-DOTA-PEG_4_-A9 where *T* tumor, *K* kidney and *B* bladder as indicated by arrows.

SPECT images of SKBR3 tumor bearing SCID mice injected with radiopeptides [^177^Lu]DOTA-A9 and [^177^Lu]DOTA-PEG_4_-A9 are presented in (Fig. 6a,b) respectively. After 3 h p.i. [^177^Lu]DOTA-A9 rapidly cleared from blood and other major organs. Radioactivity accumulation in kidneys was observed indicating prominent renal excretion of the radiopeptide. However, in case of [^177^Lu]DOTA-PEG_4_-A9 slightly increased background and higher uptake in kidney and bladder was observed after 3 h p.i. suggestive of enhanced circulation of the PEG variant. Tumor could be visualized by both the radiopeptides. Radioactivity distribution of the two radiopeptides decreased in kidneys at later time points while tumors were still visible at 24 and 48 h p.i.

## Discussion

HER2-targeted peptides are being widely investigated for visualization of HER2-positive breast cancer tumors and thereby designing the associated therapeutic action. Despite several research efforts none of the radiolabeled peptides has been able to reach the clinical settings either due to inadequate tumor uptake or low tumor-to-background ratios. Therefore, there is an ample scope for development of better HER2-targeting peptidic probes with improved biodistribution profile. Present study focused at studying the less explored HER2-targeting peptide A9.

Peptide A9 was synthesized by solid phase methodology and linked to DOTA at the N-terminal for chelation with Lu-177 radiometal. Another peptide was constructed by linking a PEG chain at the N-terminal followed by DOTA chelator. Two peptide constructs, DOTA-A9 and DOTA-PEG_4_-A9 were prepared in satisfactory yield. The recorded CD spectra of two peptides indicated no change in the structure on pegylation of the peptide. The peak at 215 nm or 218 nm is attributed to n → π* transition from lone pair on oxygen to the anti-bonding orbital of the carboxyl group whereas peak at 198 nm arises from π → π* transition^26^. Lu-177 label could be introduced in the two peptides with high efficiency. [^177^Lu]DOTA-A9 was highly hydrophilic, insertion of PEG chain between the peptide and DOTA further enhanced hydrophilicity of [^177^Lu]DOTA-PEG_4_-A9 as determined by log P values. Human serum stability measurement did not yield any release of free radiometal or formation of degradation products and > 95% intact radiopeptides were observed in radio-HPLC after 6 h incubation. Two Lu-177 labeled peptides displayed nearly similar binding affinity towards HER2-expressing cells. There are no previous studies with radiolabeled DOTA conjugated A9 peptides for drawing a comparative data. Single report of radiolabeled A9 peptide has been by Honarvar et al. where they investigated In-111 labeled DTPA conjugated peptide. [^111^In]DTPA-A9 was evaluated against HER2-expressing BT474 cells and high HER2-specific binding affinity (4.6 nM) was observed^19^. But direct comparison between the present radiopeptides and [^111^In]DTPA-A9 is precarious due to different testing cell lines and an altogether different metal-chelate.

HER2 specific binding of the two radiopeptides [^177^Lu]DOTA-A9 and [^177^Lu]DOTA-PEG_4_-A9 was confirmed by significantly lower uptake (p < 0.0005) in HER2-negative MDA-MB-231 cells in comparison to HER2-positive SKBR3 cells.

In vivo studies were further pursued with radiopeptides in SCID mice bearing SKBR3-xenograft. Maximum tumor uptake for the two radiopeptides was observed at 3 h which then decreased at 24 h and 48 h p.i. The uptake of [^177^Lu]DOTA-PEG_4_-A9 was significantly greater (p < 0.05) than that of [^177^Lu]DOTA-A9 at the three time points studied. Tumor-to-blood ratio of [^177^Lu]DOTA-A9 increased ~ twofold from 3 to 48 h whereas for [^177^Lu]DOTA-PEG_4_-A9 higher radioactivity accumulation in tumor resulted in ~ fourfold increase. Ratios were comparable for the two radiopeptides at 3 h and 24 h but were significantly higher (p < 0.005) for [^177^Lu]DOTA-PEG_4_-A9 at 48 h. Tumor-to-muscle ratio for [^177^Lu]DOTA-A9 remained nearly constant at the three time points whereas twofold increase was observed for [^177^Lu]DOTA-PEG_4_-A9 from 3 to 48 h. On comparison, tumor-to-muscle ratio for [^177^Lu]DOTA-PEG_4_-A9 was significantly higher (p < 0.005) at all the time points studied. Though both the radiopeptides rapidly cleared from blood leading to low levels of radioactivity in non-target tissues significantly higher target-to-background ratios were observed for [^177^Lu]DOTA-PEG_4_-A9. This may be due to slightly increased circulation of PEG variant in the bloodstream leading to enhanced target accumulation. [^177^Lu]DOTA-PEG_4_-A9 displayed twofold higher uptake in kidney at 3 h than [^177^Lu]DOTA-A9 indicating slower renal clearance for the PEG construct. Despite nearly similar in vitro binding affinities of the two radiopeptides, [^177^Lu]DOTA-PEG_4_-A9 demonstrated substantially better in vivo pharmacokinetics. There are no biodistribution studies reported in literature with radiolabeled A9 peptide in tumor bearing mice. Biodistribution profile of [^111^In]DTPA-A9 in normal mice at 1 h p.i. demonstrated rapid blood clearance and low radioactivity accumulation in liver, lungs, bone and gastrointestinal tract^19^. Similar clearance pattern was observed in present study for Lu-177 labeled DOTA-A9 peptides with insignificant radioactivity levels in normal tissues resulting in high tumor-to-background ratios.

Besides A9 peptide other HER2-targeting peptides have been explored and investigated. Maximum tumor uptake reported for [^111^In]DOTA(GSG)-KCCYSL (2.12 ± 0.32% ID/g, MDA-MB-435 tumors) is comparable to that of [^177^Lu]DOTA-PEG_4_-A9 (2.04 ± 0.22% ID/g). However, the tumor uptake reduced to 0.10 ± 0.02% ID/g at 24 h p.i. which is much lower than the values of presently studied radiopeptides. Rapid clearance from blood resulted in higher tumor-to-blood-ratio (10:1) at 24 h p.i. in comparison to 7.42 ± 1.11 and 8.87 ± 1.51 for [^177^Lu]DOTA-A9 and [^177^Lu]DOTA-PEG_4_-A9 respectively. Tumor-to muscle ratio however was quite low (0.5:1) at 24 h p.i. compared to 8.09 ± 2.21 and 13.3 ± 0.03 for the presently studied radiopeptides^14^. Another HER2-targeting peptide, [^111^In]DTPA-AHNP-PEG evaluated in HER2-expressing gastric cancer (GC) tumor (NCI-N87) bearing mice demonstrated tumor-to-blood ratio of 6:1 at 4 h which is higher than of currently reported radiopeptides at 3 h. Tumor-to-muscle ratio at 4 h was higher (14:1) compared to [^177^Lu]DOTA-A9 (8.42 ± 0.04) but lower than [^177^Lu]DOTA-PEG_4_-A9 (15.69 ± 2.06) at 3 h^18^.

Several Tc-99m-labeled peptides targeting HER2 receptors have also been studied. The high uptake of [^99m^Tc]HYNIC-(Ser)_3_-LTVPWY in U-87 MG tumors (2.08 ± 0.07% ID/g) at 1 h p.i. dropped to 0.04 ± 0.03% ID/g at 4 h p.i. Tumor-to-blood (3.39) and tumor-to-kidney (0.24) ratios at 4 h p.i. were comparable whereas tumor-to-muscle (3.98) ratio was much lower than the present radiopeptides. Tumor-to-blood, tumor-to-muscle and tumor-to-kidney ratios reported were much lower for [^99m^Tc]CGGG-LTVSPWY and [^99m^Tc]CSSS-LTVSPWY compared to the current radiopeptides^29^.

[^99m^Tc]HYNIC-H6F in MDA-MB-453 tumor bearing mice demonstrated tumor uptake of 2.47 ± 0.12 at 30 min p.i. which considerably dropped to 0.66 ± 0.24 and 0.21 ± 0.05 at 1 h and 2 h p.i. respectively. Recently Du et al. reported Tc-99 m-labeled pegylated versions of retro-inverso D-peptide of H6 (PEG_4_-RDH6, PEG_12_-RDH6, PEG_24_-RDH6). According to their study [^99m^Tc]PEG_4_-RDH6 had poor water solubility and exhibited high liver uptake hence prolonged PEG chains were introduced. Insertion of PEG_24_ chain ensured complete water solubility of the peptide. Enhanced hydrophilicity resulted in reduced liver and kidney uptake of [^99m^Tc]PEG_24_-RDH6 as compared to [^99m^Tc]PEG_4_-RDH6 and [^99m^Tc]PEG_12_-RDH6. Tumor-to-blood ratio of [^99m^Tc]PEG_24_-RDH6 at 2 h p.i. was comparable to that of [^177^Lu]DOTA-A9 and [^177^Lu]DOTA-PEG_4_-A9 at 24 h p.i. whereas tumor-to-muscle ratio at 2 h p.i. was comparable to that of [^177^Lu]DOTA-PEG_4_-A9 at 3 h p.i. Tumor-to-liver ratios were significantly low and kidney uptake was quite high at all the time points for [^99m^Tc]PEG_24_-RDH6 in comparison to the currently reported radiopeptides^30^. In present studies PEG_4_ chain was considered optimum as the non-pegylated peptide DOTA-A9 had no solubility issues and [^177^Lu]DOTA-A9 did not exhibit high uptake or retention in liver/kidney. The PEG chain was introduced with the aim of increasing the blood retention of the radiopeptide and increase the tumor uptake. Enhanced uptake in tumor could be achieved with PEG_4_ chain but higher uptake in kidney was also observed in comparison to the non-pegylated radiopeptide. An increase in PEG length is likely to further increase the uptake in the kidney and increase in molecular size may also result in enhanced liver uptake hence higher PEG chains were not considered in the present study.

SPECT images of [^177^Lu]DOTA-A9 and [^177^Lu]DOTA-PEG_4_-A9 demonstrated rapid washout from major organs. [^177^Lu]DOTA-PEG_4_-A9 demonstrated higher uptake in kidneys as well as slightly higher radioactivity accumulation in tumor as compared to [^177^Lu]DOTA-A9 matching the biodistribution results. The present work can be further extended in metastatic and other HER2 models in future to further explore and establish the probe potential.

The PEG construct, [^177^Lu]DOTA-PEG_4_-A9 has shown encouraging in vivo profile but clinical transformation would require further improvement in tumor uptake and retention. Altered engineering of A9 peptide with longer PEG chain or a lipophilic linker can probably result in reformed performance.

## Conclusion

In conclusion, we describe here synthesis and comparative evaluation of HER2-targeting DOTA-A9 peptide and its PEG variant. Two peptide constructs could be synthesized with high purity using robust solid phase technique and radiolabeled with Lu-177 in high yield. [^177^Lu]DOTA-A9 and [^177^Lu]DOTA-PEG_4_-A9 exhibited high binding affinity towards breast cancer cells in low nanomolar range. Biodistribution studies demonstrated low levels of peptidic probes in blood and other non-target tissues. Better pharmacokinetics and target-to-background ratios were observed with pegylated peptide probe in comparison to the non-pegylated probe. This study provides encouraging results for further exploring newer A9 peptide variants.

## Methods

### Materials

9-Fluorenylmethoxycarbonyl (Fmoc)-protected amino acids, Fmoc-NH-PEG_4_-COOH, NovaSyn TGR resin and other reagents and solvents used for peptide synthesis were purchased from Novabiochem (Germany). Bifunctional chelator DOTA-tris(t-Bu)ester was procured from Chematech (France). Methanol and acetonitrile were of HPLC grade. All other solvents and chemicals were purchased from Sigma-Aldrich, USA. Matrix assisted laser desorption/ionization-time of flight MALDI-TOF mass spectrometry analysis was carried out at Tata Institute of Fundamental Research (TIFR). Peptides were purified by semi preparative RP-HPLC (JASCO, Japan) connected with JASCO-PU-2086 Plus, intelligent prep pump, JASCO UV-2075 Plus absorption detector [Megapak Sil C18-10 column (7.5 × 250 mm)]. Analytical RP-HPLC was performed using JASCO PU2080 Plus dual pump HPLC system (Japan) with a JASCO 2075 Plus tunable absorption detector and a Gina Star radiometric detector system [C_18_ reversed phase HiQ Sil column (5 μm, 4 × 250 mm)]. Mobile phase gradient used for analysis: 0–28 min, 90–10% A; 28–30 min, 10% A; and 30–32 min, 10–90% A; flow of 1 mL/min; 0.1% TFA in H_2_O (A) and 0.1% TFA in acetonitrile (B). For purification similar gradient system was used with a flow rate of 2 mL/min.

Radioactivity measurements were done using a well-type NaI(Tl) gamma counter (Raytest, Germany). Human breast carcinoma, SKBR3 cell line was procured from the National Centre for Cell Sciences (NCCS) Pune, India. Female SCID mice were purchased from Laboratory Animal Facility (LAF) of Advanced Centre for Treatment, Research and Education in Cancer (ACTREC), Navi Mumbai, India. Animal studies were approved by the Institutional Animal Ethics Committee (IAEC) of BARC and all animal experiments were carried out in strict compliance with the institutional guidelines following the relevant national laws related to the conduct of animal experimentation. In vivo SPECT imaging studies were performed on the U-SPECT + (M/s MI Labs, The Netherlands). The study is reported in accordance with ARRIVE guidelines.

### Peptide synthesis

Peptides, DOTA-A9 and DOTA-PEG_4_-A9 were synthesized manually by Fmoc solid-phase peptide synthesis. The protected peptide sequence Gln(Trt)-Asp(OtBu)-Val-Asn(Trt)-Thr(tBu)-Ala-Val-Ala-Trp(Boc)-NH_2_ was constructed on NovaSyn TGR resin. Amino acid couplings were performed by activation of Fmoc-amino acids (3 eq.) with *O*-(7-azabenzotriazol-l-yl)-*N*,*N*,*N*′,*N*′-tetramethyluroniumhexafluorophosphate (HATU) (3 eq.) and *N*, *N’-*diisopropylethylamine (DIPEA) (6 eq.) in dimethylformamide (DMF) followed by gentle rotation for 120 min. Fmoc-deprotection was carried out with 20% piperidine in DMF (v/v, 2 × 10 min). Coupling reactions were monitored by the colorimetric trinitrobenzenesulphonic acid (TNBS) test. For PEGylation, N-terminus of the protected peptide was reacted with Fmoc-NH-PEG_4_-COOH (19 atoms) in presence of HATU and DIPEA. The chelator was incorporated at N-terminus by coupling of DOTA-tris (t-Bu)ester (3 eq.) in presence of HATU and DIPEA. The peptide was finally cleaved from the resin with a cocktail mixture of trifluoroacetic acid (TFA)/triisopropylsilane (TIPS)/H_2_O (95/2.5/2.5, v/v) wherein protecting groups were also removed. The crude peptide was precipitated by addition of diethyl ether. The precipitate obtained was dissolved in water and washed with ether (× 3). Crude peptide was purified using semi-preparative HPLC and lyophilized to obtain white fluffy powder.

MALDI-TOF (DOTA-A9); calcd for C_60_H_93_N_17_O_21_, 1388.48; found, 1388.517.

MALDI-TOF (DOTA-PEG_4_-A9); calcd for C_73_H_118_N_18_O_27_, 1679.82; found, 1679.682.

### Circular dichroism (CD)

CD spectra were recorded using a Jasco J-815 CD spectrometer and a quartz flow cell of 1 mm path length. Measurements were carried out at room temperature (25 °C) in the far-ultraviolet region (260–190 nm). Peptide samples were dissolved in HPLC grade water in concentration of 100 µM. Each spectrum was collected at the scan rate 50 nm/min with a band width of 1 nm which was averaged over 5 scans. The baseline (water) was subtracted from the spectra.

### Radiochemistry

Peptides DOTA-A9 and DOTA-PEG_4_-A9 were radiolabeled with [^177^Lu]LuCl_3_ following the reported protocol^25^. Briefly, sodium acetate buffer (200 µL, 1.5 M, pH 5) was added to an aqueous suspension of peptides (50 µg) followed by addition of [^177^Lu]LuCl_3_ (110 MBq). The reaction mixture was incubated at 80 °C for 10 min. Radiolabeling yield was determined by analytical RP-HPLC and radiochemical yield was determined by paper chromatography.

Lipophilicity of [^177^Lu]DOTA-A9 and [^177^Lu]DOTA-PEG_4_-A9 was assessed by determining the partition coefficient (Log P_o/w_) of peptide tracers in immiscible n-octanol/water solvent system following the earlier reported procedure^26^.

For serum stability studies blood was allowed to clot (1 h) followed by centrifugation (2300×*g*, 10 min). Measurements were performed by incubation of radiopeptides (25 µL, 3.7 MBq) with human serum (500 μL, pH 7.4) at 37 °C. After 6 h incubation acetonitrile (500 μL) was added to the sample for protein precipitation. Subsequently the sample was centrifuged, supernatant was collected and radiochemical purity was analyzed by RP-HPLC.

### In vitro cell studies

SKBR3 (HER2-positive) and MDA-MB-231 (HER2-negative) cells were grown to 70–80% confluence in Dulbecco’s Modified Eagle’s Medium (DMEM) containing 10% fetal bovine serum (Invitrogen Carlsbad, CA) and 1% antibiotic/antimycotic formulation. Cells were harvested and seeded (1 × 10^6^) in 12-well tissue culture plates and incubated overnight at 37 °C in a humidified atmosphere containing 5% CO_2_. Subsequently, cells were incubated with [^177^Lu]DOTA-A9 and [^177^Lu]DOTA-PEG_4_-A9 at 37 °C for 2 h. The assays were carried out in triplicate. After incubation, cells were washed twice with cold phosphate buffer saline (PBS) and solubilized with 1 N NaOH (1 mL). Radioactivity associated with cells was measured in NaI (Tl) gamma counter. Inhibition studies were carried out by pre-incubation of cells with unlabeled A9 peptide.

Spheroids of SKBR3 and MDA-MB-231 cells were produced using Ultra-Low attachment microplates (Corning, USA) (Supplementary Fig. S3). For both the cell types 2000 cells were seeded per well and cultured for 7 days. After 7 days spheroids were incubated with radiopeptides [^177^Lu]DOTA-A9 and [^177^Lu]DOTA-PEG_4_-A9 (~ 10,000 cps, 40 ng) for 2 h at 37 °C. Spheroid washing and counting procedure was similar to that mentioned above for the cell binding assay. Images of the spheroids were captured by phase contrast microscopy using inverted light microscope (10 × magnification) (Nilpa, India).

The binding affinity of [^177^Lu]DOTA-A9 and [^177^Lu]DOTA-PEG_4_-A9 was determined by the saturation radioligand binding assay in HER2 over expressing SKBR3 cells. 1 × 10^6^ cells/well were seeded in cell culture plates and incubated at 37 °C. Cells were treated with series of increasing concentration of the radiolabeled peptide (4–200 nM) and incubated at 37 °C for 60 min. For obtaining non-specific binding, cells were co-incubated with radiolabeled formulations along with unlabeled DOTA-A9 peptide. On completion of incubation, cells were washed with the ice-cold phosphate buffer saline (PBS). Cells with bound radioactivity were harvested with 1 N NaOH (1 mL) solution (at 37 °C for 3 min) and counted in a gamma counter. K_d_ value was calculated by the nonlinear regression algorithm (GraphPad Prism version 7.0). The B_max_ value (nM) was converted to the number of HER2 receptors per cell.

### SPECT imaging studies

SKBR3 tumour bearing female SCID mice were injected with [^177^Lu]DOTA-A9 and [^177^Lu]DOTA-PEG_4_-A9 (150 μL, 37 MBq) intravenously via the tail vein (n = 3). SPECT image acquisition was performed using pre-clinical scanner at different time points (3, 24 and 48 h) after anesthetizing the animals with isoflurane. SPECT images were acquired for 15 min using SPECT scanner and the data was reconstructed. The SPECT data was analyzed using PMOD v4.003.

### Biodistribution studies

Biodistribution studies of peptide tracers, [^177^Lu]DOTA-A9 and [^177^Lu]DOTA-PEG_4_-A9 were carried out in female SCID mice bearing human breast carcinoma, SKBR3 tumor xenografts. Tumor bearing mice (n = 3) were intravenously injected with peptide tracers (0.5 μg, 150 μL, 3.7 MBq) into the tail vein. Animals were sacrificed and dissected at 3 h, 24 h and 48 h post injection (p.i.). The radioactivity associated with each tissue was counted in a NaI (Tl) flat geometry detector and expressed as percentage injected dose per gram of tissue [% ID/g, mean ± standard deviation, n = 3/radiotracer].

Statistical analysis using the paired two-tailed Student’s t-test was performed to compare uptake values in tumor and major organs between [^177^Lu]DOTA-A9 and [^177^Lu]DOTA-PEG_4_-A9 animal groups; values of p < 0.05 were considered statistically significant.

## Supplementary Information

Supplementary Figures.

## Data Availability

The datasets generated during and/or analysed during the current study are available from the corresponding author on reasonable request.
