# Author Correction: Synthesis and comparative evaluation of ^177^Lu-labeled PEG and non-PEG variant peptides as HER2-targeting probes

**DOI:** 10.1038/s41598-022-23594-y

**Published:** 2022-11-08

**Authors:** Amit Kumar Sharma, Rohit Sharma, Kusum Vats, Haladhar Dev Sarma, Archana Mukherjee, Tapas Das, Drishty Satpati

**Affiliations:** 1 Radiopharmaceuticals Division, Bhabha Atomic Research Centre, Mumbai, India; 2 Homi Bhabha National Institute, Mumbai, India; 3 Radiation Biology and Health Sciences Division, Bhabha Atomic Research Centre, Mumbai, India

Correction to: *Scientific Reports* 10.1038/s41598-022-19201-9, published online 20 September 2022

The original version of this Article contained an error in Reference 22, which was incorrectly given as:

Luca, S. D., Verdoliva, V. & Saviano, M. Peptide ligands specifically targeting HER2 Receptor and the role played by a synthetic model system of the receptor extracellular domain: Hypothesized future perspectives. *J. Med. Chem.* **63**, 15333–15343 (2020).

The correct reference is listed below:

De Luca, S., Verdoliva, V. & Saviano, M. Peptide ligands specifically targeting HER2 Receptor and the role played by a synthetic model system of the receptor extracellular domain: Hypothesized future perspectives. *J. Med. Chem.* **63**, 15333–15343 (2020).

The original Article has been corrected.

