# Supplementary material for: Synthesis and comparative evaluation of 177Lu-labeled PEG and non-PEG variant peptides as HER2-targeting probes
Source: Sci Rep. 2022 Sep 20;12:15720. doi: 10.1038/s41598-022-19201-9 (PMC9489682; doi:10.1038/s41598-022-19201-9)
Supplement: Supplementary file 1 — Supplementary Figures. [file 41598_2022_19201_MOESM1_ESM.docx]

**Supplementary Information**

**Synthesis and Comparative Evaluation of ^177^Lu-labeled PEG and non-PEG variant peptides as HER2-targeting probes**

Amit Kumar Sharma^1,2^, Rohit Sharma^1,2^, Kusum Vats^1,2^, Haladhar Dev Sarma^3^,

Archana Mukherjee^1,2^, Tapas Das^1,2^, Drishty Satpati^*1,2^

^1^ Radiopharmaceuticals Division,

Bhabha Atomic Research Centre, Mumbai, India;

^2^Homi Bhabha National Institute, Mumbai, India;

^3^Radiation Biology and Health Sciences Division,

Bhabha Atomic Research Centre, Mumbai, India

Author for Correspondence: Drishty Satpati, PhD

Email: [drishtys@barc.gov.in](mailto:drishtys@barc.gov.in)

Ph: 91-22-25590748

Fax: 91-22-25505151


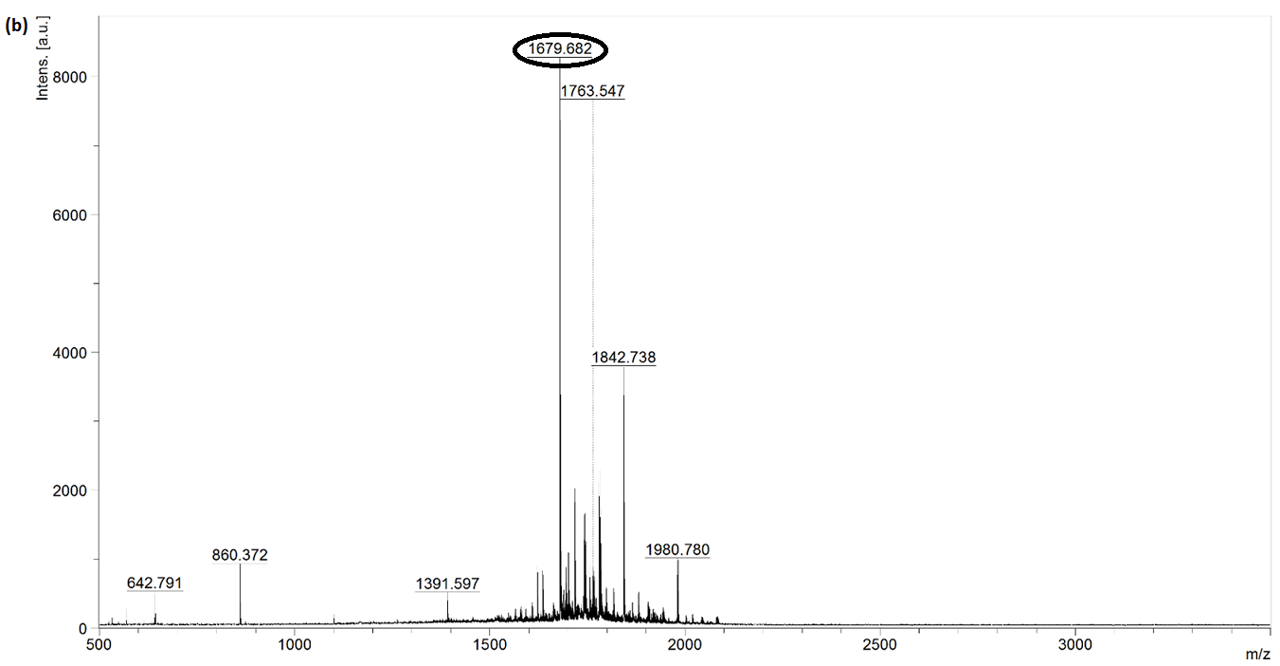

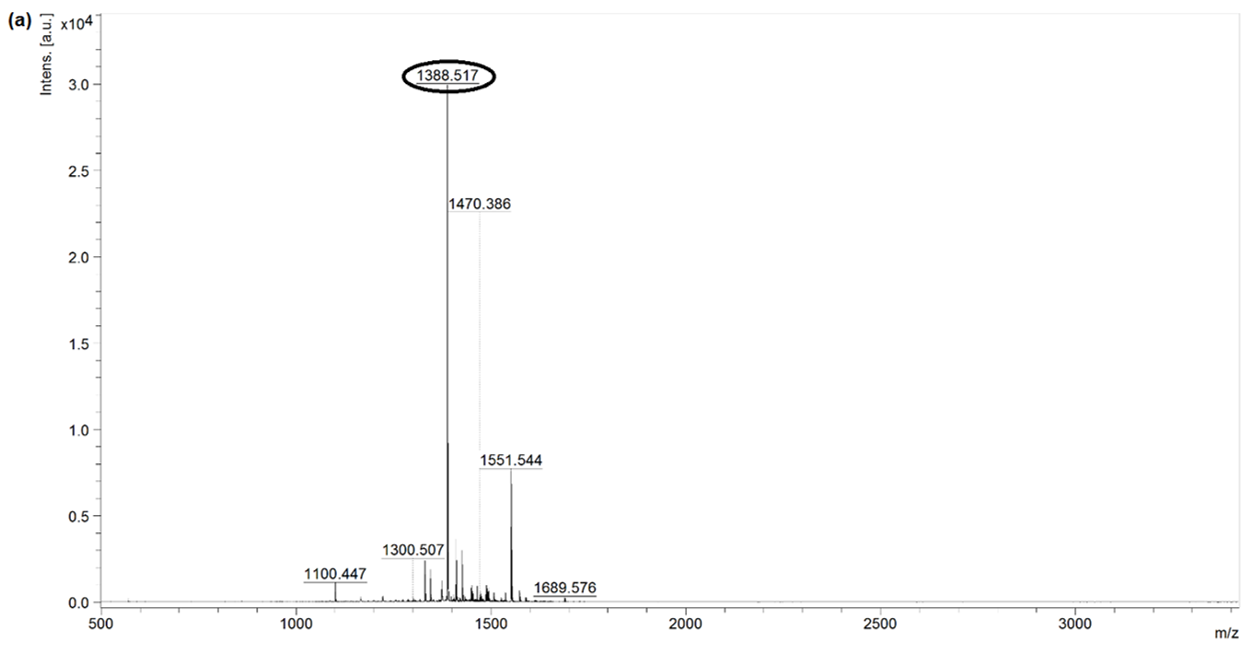


**Supplementary Figure S1.** MALDI-TOF MS spectra (a) DOTA-A9 and (b) DOTA-PEG_4_-A9

**Supplementary Figure S2.** CD spectrum of (a) DOTA-A9 and (b) DOTA-PEG_4_-A9 peptides


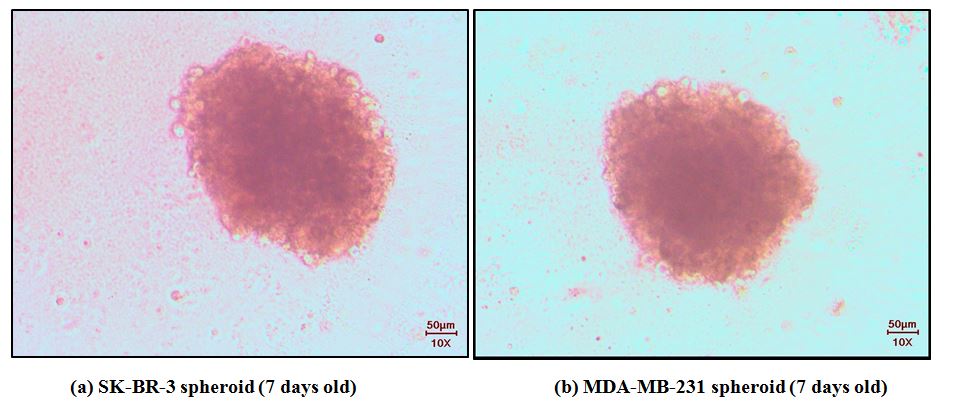


**Supplementary Figure S3.** Morphology in ultra-low attachment plates of (a) SKBR-3 spheroid and (b) MDA-MB-231 spheroid
